# Pilot Validation of AI-Derived Features for Prognostic Models in Geriatric Oncology

**DOI:** 10.1093/geroni/igaf122.4174

**Published:** 2025-12-31

**Authors:** Alaa Albashayreh, W Nick Street, Stephanie Gilbertson-White

**Affiliations:** University of Iowa, Iowa City, Iowa, United States; University of Iowa, Iowa City, Iowa, United States; University of Iowa, University of Iowa, Iowa, United States

## Abstract

Accurate survival prediction for older adults with lung cancer is critical for guiding supportive care, yet standard models often underutilize valuable information in clinical notes. The objective of this study was to determine if a prognostic model integrating features from unstructured clinical notes could more accurately predict survival in older adults with lung cancer than a model using structured data alone. We studied 1,391 patients (≥60 years at time of diagnosis) with lung cancer, analyzing 19,377 clinical encounters. We compared a baseline survival model using structured electronic health record (EHR) data to an enriched model that integrated previously validated natural language processing (NLP)-derived features, including functional status. Models were evaluated for 12-month mortality using AUC and C-Statistic. Thirty-eight percent of patients presented with metastatic disease. The baseline prediction model achieved good performance (AUC: 0.740; C-Statistic: 0.697). The enriched model demonstrated statistically significant improvement (AUC: 0.768 vs. 0.740; C-Statistic: 0.717 vs. 0.697; DeLong’s test p<.0001) with well-calibrated predictions and effective risk stratification into distinct prognostic groups. Feature analysis confirmed NLP-derived functional status and longitudinal chemotherapy timing were key predictors, with functional status ranking among the top three most important features. Clinical notes contain crucial prognostic information not captured in structured data. Leveraging NLP to extract this narrative data offers a powerful pathway to more accurate survival prediction, which could enable better identification of high-risk patients to improve supportive care interventions.

